# The rice XA21 ectodomain fused to the Arabidopsis EFR cytoplasmic domain confers resistance to *Xanthomonas oryzae* pv. *oryzae*

**DOI:** 10.7717/peerj.4456

**Published:** 2018-05-09

**Authors:** Nicholas C. Thomas, Nir Oksenberg, Furong Liu, Daniel Caddell, Alina Nalyvayko, Yen Nguyen, Benjamin Schwessinger, Pamela C. Ronald

**Affiliations:** 1 Department of Plant Pathology and the Genome Center, University of California, Davis, Davis, CA, USA

**Keywords:** Rice, XA21, EFR, Disease resistance, Xanthomonas, Immune receptor, Chimeric receptor

## Abstract

Rice (*Oryza sativa*) plants expressing the XA21 cell-surface receptor kinase are resistant to *Xanthomonas oryzae* pv. *oryzae (Xoo)* infection. We previously demonstrated that expressing a chimeric protein containing the ELONGATION FACTOR Tu RECEPTOR (EFR) ectodomain and the XA21 endodomain (EFR:XA21) in rice does not confer robust resistance to *Xoo*. To test if the XA21 ectodomain is required for *Xoo* resistance, we produced transgenic rice lines expressing a chimeric protein consisting of the XA21 ectodomain and EFR endodomain (XA21:EFR) and inoculated these lines with *Xoo*. We also tested if the XA21:EFR rice plants respond to a synthetic sulfated 21 amino acid derivative (RaxX21-sY) of the activator of XA21-mediated immunity, RaxX. We found that five independently transformed XA21:EFR rice lines displayed resistance to *Xoo* as measured by lesion length analysis, and showed that five lines share characteristic markers of the XA21 defense response (generation of reactive oxygen species and defense response gene expression) after treatment with RaxX21-sY. Our results indicate that expression of the XA21:EFR chimeric receptor in rice confers resistance to *Xoo*. These results suggest that the endodomain of the EFR and XA21 immune receptors are interchangeable and the XA21 ectodomain is the key determinant conferring robust resistance to *Xoo*.

## Introduction

Plant cell-surface immune receptors confer defense against pathogen infection. Cell-surface mediated immunity in plants is mainly conferred by receptor-like proteins (RLPs) and receptor-like kinases (RLKs) that recognize pathogen associated molecular patterns (Jones & Dangl, 2006; Macho & Zipfel, 2014). Three well-studied cell-surface RLKs that confer resistance to bacterial pathogens include FLAGELLIN SENSING2 (FLS2; At5G6330) (Gómez-Gómez & Boller, 2000), EF-TU RECEPTOR (EFR; At5g20480) (Zipfel et al., 2006) from *Arabidopsis* and XA21 (U37133) from *Oryza longistaminata* (Song et al., 1995). The identification of the microbial molecules recognized by these three receptors have enhanced in depth characterization of their functional properties. The FLS2 receptor binds the flg22 peptide derived from bacterial flagellin (Felix et al., 1999; Gómez-Gómez & Boller, 2000; Chinchilla et al., 2006). EFR recognizes the elf18 peptide derived from the bacterial Elongation Factor Thermo-unstable protein (EF-Tu) (Kunze et al., 2004; Zipfel et al., 2006). XA21 recognizes the sulfated required for activation of Xa21-mediated immunity X (RaxX) protein produced by *Xanthomonas oryzae* pv. *oryzae* (*Xoo*; Pruitt et al., 2015). Although these receptors specifically recognize different molecules, they share similar domain structures including ectodomains containing leucine rich repeats and endodomains containing intracellular kinases of the non-arginine aspartate (non-RD) class (Dardick & Ronald, 2006).

Domain swap studies between cell-surface receptors have led to the hypothesis that the nature of the endodomains is the primary determinant dictating the specific disease resistance outcome. For example, studies of a chimeric receptor generated by fusion of the *Arabidopsis* BRASSINOSTEROID-INSENSITIVE1 (BRI1) receptor, which recognizes brassinosteroid hormones, to the XA21 endodomain (BRI1:XA21) (Li & Chory, 1997) indicated that the chimeric receptor could be activated by brassinosteroid treatment. Rice cells expressing BRI1:XA21 (NRG-1 in the original publication) and treated with brassinosteroid initiated cell death, produced reactive oxygen species (ROS), and expressed stress-related genes. The stress-related symptoms were attributed to the activation of the XA21 endodomain because the full-length BRI1 receptor does not induce the same stress-related symptoms as BRI1:XA21 (He et al., 2000). These results suggested that the XA21 endodomain was activated upon BRI1 recognition of brassinosteroid and that the specific type of response was most consistent with the response mediated by the XA21 endodomain and not the BRI1 ectodomain.

Another chimera study compared the responses of receptors consisting of ectodomain and endodomain exchanges between EFR and WAK1. WAK1 recognizes oligogalacturonides (OGs) released from damaged plant cell walls (Decreux & Messiaen, 2005; Decreux et al., 2006; Cabrera et al., 2008). Elf18 treated wild-type plants and OG treated plants expressing a WAK1:EFR (WEG) chimeric RLK both produced ROS, ethylene and expressed the EFR-induced genes (At3g22270 and At4g37640) while EFR:WAK1 (EWAK) expressing plants did not. Instead, EWAK plants retained WAK1-like responses by producing ROS but not ethylene in response to OGs (Ferrari et al., 2008; Brutus et al., 2010). WEG and EWAK responses were therefore most consistent with the response conferred by the respective endodomain portion of each fusion protein. Another study showed that fusing the XA21 endodomain to the fungal chitin RLP CEBiP (Kaku et al., 2006) (CRXA-1, and CRXA-3 in the original publication) conferred a more robust immune response to fungal infection by *Magnaporthe oryzae* than when expressing or overexpressing CEBiP alone (Kishimoto et al., 2010). These results suggested that the XA21 endodomain was responsible for conferring the enhanced immune response to *M. oryzae*. Together, these studies indicate that the endodomain of several immune receptors dictate the specific signaling events that lead to disease resistance in whole plants or defense responses in plant cells. These studies also suggest chimeras carrying the XA21 endodomain, when treated with the appropriate ligand, can initiate an immune response similar to that mediated by the full-length receptor XA21.

To further explore the function and specificity of the XA21 endodomain and ectodomain, we previously generated transgenic rice lines expressing EFR, tagged with green fluorescent protein (EFR:GFP), or a chimeric EFR:XA21 protein, consisting of the EFR ectodomain and the XA21 transmembrane and intracellular domain, tagged with GFP (EFR:XA21:GFP). The maize ubiquitin promoter drove expression for both *EFR:GFP* and *EFR:XA21:GFP* transgenes (Schwessinger et al., 2015a). Both *EFR:GFP* and *EFR:XA21:GFP* rice plants were susceptible to *Xoo* strain PXO99A and conferred partial resistance to weakly virulent strains, which suggested EF-Tu from *Xoo* was still recognized by *EFR:GFP* rice (Schwessinger et al., 2015a). These studies suggested that although both receptors were capable of recognizing EF-Tu, they were still unable to initiate a robust immune response to PXO99A. As noted in the paper’s discussion, these results were counterintuitive based on earlier domain swap studies that indicated that the endodomain dictates immune signaling and disease resistance (He et al., 2000; Brutus et al., 2010; Albert et al., 2010; Kishimoto et al., 2010).

Although it is unclear why the EFR and EFR:XA21 study conflicted with findings from previous chimeric receptor studies, there are several possibilities to explain these discrepancies. In the case of the EFR:WAK1 and WAK1:EFR study, it could be that the type of kinase domain dictated the distinct signaling mediated by each chimeric receptor because the WAK1 and EFR kinase domains belong to different kinase classes. The WAK1 kinase domain contains an arginine (R) aspartate (D) motif while the EFR kinase domain is non-RD, as described above. The non-RD kinases are almost always associated with immune responses in plants and animals and are likely regulated differently than RD kinases (Dardick & Ronald, 2006; Ronald & Beutler, 2010; Dardick, Schwessinger & Ronald, 2012). Thus, the presence of the non-RD domain may dictate an immune response when appropriately activated and the presence of the RD domain may specify a WAK1-like response.

For both BRI1:XA21 and CEBiP:XA21 studies, it is possible that the origin of the kinase domain from XA21 was less important than the fact that the kinase belonged to the non-RD class. For example, it is unclear if fusing BRI1 or CEBiP to other non-RD kinases, such as the kinases from EFR or OsFLS2, would have produced similar results (Takai et al., 2008).

Previous studies have shown that the XA21 ectodomain plays a critical role in the immune response. For example, the *Xa21D* paralog, which lacks a transmembrane and intracellular domain, confer partial resistance to *Xoo* (Wang et al., 1998). Unlike *Xa21*, *Xa21D* only encodes an ectodomain that is nearly identical to the XA21 ectodomain, differing only in 15 amino acid residues compared to the XA21 ectodomain. Similarly, expression of a catalytically inactive variant of XA21, carrying a mutation in the catalytic domain of the kinase (K736E), in rice maintained partial resistance to *Xoo* (Andaya & Ronald, 2003). Together, these studies indicate that the XA21 ectodomain is sufficient to confer partial resistance to *Xoo,* even in the absence of a functional kinase domain.

To further explore the function and importance of the XA21 ectodomain, we generated transgenic rice lines expressing a chimeric protein containing the XA21 ectodomain fused to the EFR transmembrane and intracellular domain, tagged with GFP (XA21:EFR:GFP) (Holton et al., 2015). We found that *XA21:EFR:GFP* rice display robust resistance to *Xoo* strain PXO99A. We also show that *XA21:EFR:GFP* was specifically activated by RaxX as measured by bacterial infection, defense response gene expression and ROS production (Pruitt et al., 2015; Schwessinger et al., 2015b; Wei et al., 2016). These results indicate that the XA21 ectodomain and its recognition of RaxX specify robust resistance to *Xoo* even in the absence of the XA21 endodomain.

## Materials and Methods

### Plant material and methods

Rice seeds were germinated on water-soaked filter paper for 5–7 days at 28 °C and then transplanted into 2.6-l pots. Plants were grown in an approximately 80/20 (sand/peat) soil mixture in an environmentally-controlled greenhouse with temperature set between 28 and 30 °C with 75–80% humidity.

### Transgenic rice production

The *Xa21:EFR:GFP* (XA21 aa residues 1–650 fused to EFR aa residues 650–1,031) binary vector used in rice transformation was described previously (Holton et al., 2015). Transgenic Kitaake plants expressing the *Xa21:EFR:GFP* transgene, under control of the maize ubiquitin promoter, were generated by the UC Davis Plant Transformation Facility as described previously (Hiei et al., 1994). pCAMBIA1300 binary vectors carrying the *Xa21:EFR:GFP* construct were transformed into Kitaake calli by Agrobacterium-mediated transformation. Regenerated plants were selected on hygromycin. The presence of the transgene was confirmed in each generation by PCR using transgene-specific primers (Table S1).

### Bacterial infection of rice plants

*Xoo* isolates PXO99A, PXO99A*ΔraxX*, and PXO99A*ΔraxX(raxX*) (Pruitt et al., 2015) were plated on peptone sucrose agar plates for three days. *Xoo* was suspended in water to approximately 5 × 10^8^ colony forming units (CFU)/mL for inoculation. Greenhouse-grown plants were transported into environmentally-controlled growth chambers at the four-week-old stage. Chamber conditions were set to 26 °C, 85% humidity with 12 h light/dark cycles. Plants were acclimated to the chamber conditions for two to three days before scissor inoculation (Kauffman et al., 1973).

### Segregation analysis

The presence of each transgene was identified using PCR genotyping using genomic DNA templates and transgene-specific primers (Table S1). Chi-square tests were used to determine possible multiple transgene insertions.

### Gene expression analysis by qRT-PCR

Total RNA was extracted from detached leaves frozen in liquid nitrogen and powdered using a Qiagen tissuelyser. RNA was extracted from powdered tissue using TRI Reagent and precipitated with isopropanol. RNA was DNase treated using the TURBO DNase kit from Life Technologies, Carlsbad, CA USA. RNA concentrations were normalized to the lowest sample concentration in each experiment. cDNA was synthesized from 2 μg of total RNA using the High Capacity cDNA Reverse Transcription Kit by Life Technologies, Carlsbad, CA USA. Gene expression changes were determined by ΔΔCt method (Livak & Schmittgen, 2001) normalizing gene expression to Actin (*LOC_Os03g50885*) and using mock treated samples as the reference for stress gene expression. Quantitative real time PCR (qRT-PCR) was performed using a Bio-Rad CFX96 Real-Time System coupled to a C1000 Thermal Cycler (Bio-Rad, Hercules, CA USA) using the Bio-Rad SsoFast EvaGreen Supermix. qRT-PCR primer pairs used are described in Table S1. qRT-PCR reactions were run for 40 cycles with annealing and amplification at 62 °C for 5 s and denaturation at 95 °C for 5 s. Single melting curves were observed for all primer pairs used indicating negligible off-target amplification.

### Western blot analysis for protein expression

Anti-GFP (Santa Cruz Biotech, Santa Cruz, CA USA) was used to detect EFR:GFP, EFR:XA21:GFP, XA21:GFP, and XA21:EFR:GFP. Secondary anti-mouse antibodies (Santa Cruz Biotech) conjugated to horseradish peroxidase were used in combination with chemiluminescence substrates (Thermo, Waltham, MA USA) to detect proteins on a Biorad ChemiDoc.

### Reactive oxygen species production

Leaves of three- to four-week-old rice plants were cut longitudinally along the mid vein and then into 1–1.5 mm thick pieces. Leaf pieces were floated on sterile water overnight. The following morning, two leaf pieces were transferred into one well of a 96-well white plate containing 100 μl elicitation solution (20 μM L-012 (Wako Chemicals, Neuss, Germany), 2 μg/mL HRP (Sigma, St. Louis, MO, USA)). A total of 500 nM of elf18 (*Escherichia coli*) or RaxX21-sY peptides were used for treatments. ROS production was measured for 0.5 s per reading with a high sensitivity plate reader (TriStar; Berthold, Bad Wildbad, Germany).

## Results

### Transgenic rice expressing the XA21:EFR chimeric receptor display robust resistance to *Xoo*

We produced transgenic rice lines expressing an *Xa21:EFR:GFP* chimeric construct to test whether the XA21 ectodomain confers resistance to *Xoo* when fused to the EFR cytoplasmic domain. This construct encodes the XA21 ectodomain (XA21 residues 1–650) fused to the EFR transmembrane, juxtamembrane, and cytoplasmic domain (EFR residues 651–1,031) with a carboxyl-terminal GFP fusion (Holton et al., 2015) expressed under the maize ubiquitin promoter. We generated 10 independent transgenic *T*_0_ lines and inoculated the plants with *Xoo* using a leaf clipping method followed by lesion length measurements, which allows a comparable but more rapid assessment of resistance than bacterial population counting. We found that eight of these lines (lines 2, 3, 4, 5, 6, 7, 9, and 10) displayed enhanced resistance to *Xoo* compared with the Kitaake parent line (Fig. S1).

**Figure 1:**
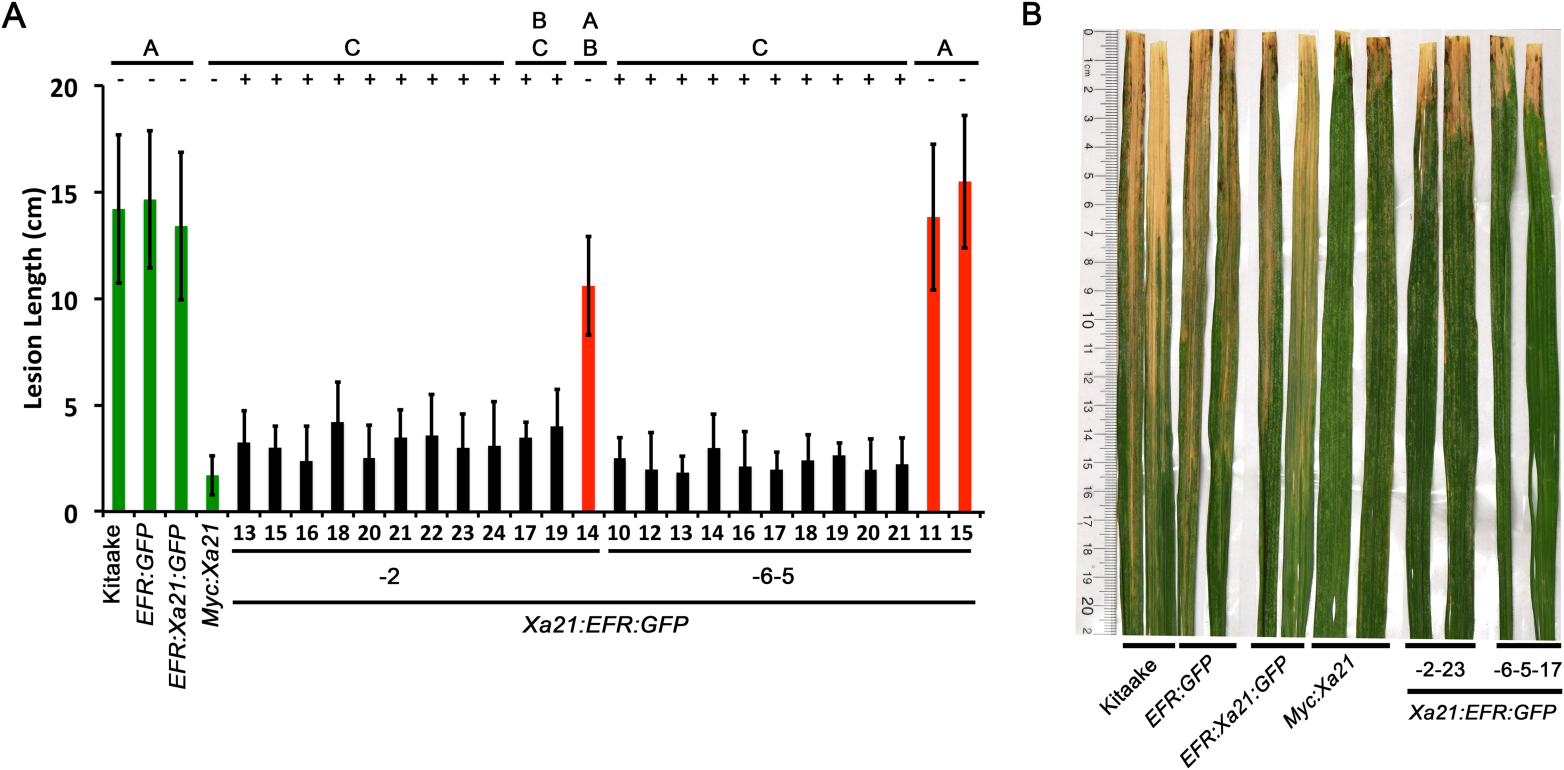
Rice expressing *Xa21:EFR:GFP* are resistant to *Xoo* infection. (A) The bar graph represents the average lesion length observed on rice plants infected with *Xoo*. Control lines used were Kitaake, *EFR:GFP*, *EFR:XA21:GFP*, and *Myc:XA21* rice (green bars). Experimental samples include individuals PCR positive for the *Xa21:EFR:GFP* transgene (black bars) from line 2 and 6 and PCR negative individuals (red bars). Five-week-old greenhouse-grown plants were scissor inoculated with PXO99A (5 × 10^8^ CFU/mL) and disease lesions were scored approximately two weeks post inoculation. Error bars represent standard deviation from the mean lesion length. Mean lesion lengths are the average of lesion measurements from individual leaves from the same plant (*n* ≥ 3). Black lines and letters above the graph represent statistical groupings using the Tukey–Kramer HSD test. Different letters indicate significant differences (*p* < 0.05). This experiment was repeated at least three times with similar results. (B) Photograph of select leaves from the same experiment in A. The photograph shows Kitaake, *EFR:GFP*, *EFR:Xa21:GFP*, *Myc:Xa21*, *Xa21:EFR:GFP* individual -2-23, and -6-5-17 leaves infected with *Xoo* and was taken approximately two weeks after inoculation. Nicholas Thomas provided the photograph.

To assess if the resistance phenotype was transmitted to the next generation, we self-pollinated five of the eight *T*_0_ lines (lines 2, 4, 5, 6, 7) and collected *T*_1_ seed. These *T*_1_ plants, as well as rice plants expressing and lacking *Xa21* as controls, were inoculated with *Xoo* and assessed for resistance by measuring the lengths of disease-induced lesions. We observed that *T*_1_ individuals that were PCR positive for the transgene in lines 2, 4, 5, and 6 co-segregated with resistance to *Xoo* (PCR positive to negative ratios 8:4, 21:0, 8:7, and 16:5, respectively). Lesion length averages were approximately 5 cm in resistant individuals compared to approximately 13 cm for susceptible controls (Fig. 1 and Fig. S2). All *T*_1_ individuals from line 4 were PCR positive for *Xa21:EFR:GFP* (21:0) which could have been from multiple transgene insertions (*X^2^* (1) = 1.4, *p* = 0.24) and were resistant to *Xoo*. All *T*_1_ individuals from line 7 were also PCR positive for the *Xa21:EFR:GFP* transgene. However, these plants showed varying degrees of resistance (Fig. S2).

To determine if *Xoo* resistance in *Xa21:EFR:GFP* plants is mediated through XA21 perception of RaxX, we infected two different T1 progeny from two different -2 lines (-2-13 and -2-19) and T2 progeny from line -6-5-1 with wild-type PXO99A, PXO99A Δ*raxX* mutants (Δ*raxX*) that evade full-length XA21-medaited immunity, and Δ*raxX* strains complemented with *raxX* (Δ*raxX(raxX)*) that do no evade XA21-mediated immunity (Pruitt et al., 2015). We found that Kitaake plants were susceptible to all strains used (average lesion lengths approximately 20 cm), whereas *Xa21:GFP* control plants were susceptible to Δ*raxX* infections with approximately 19 cm average lesion lengths and resistant to WT PXO99A and complemented Δ*raxX(raxX*) strains with approximately 7 cm average lesion lengths consistent with previous findings (Pruitt et al., 2015). For segregants carrying the *Xa21:EFR:GFP* transgene, plants were significantly more resistant to WT PXO99A, with approximately 5 cm average lesion lengths, compared to Δ*raxX* infections that developed lesions approximately 17 cm long. *Xa21:EFR:GFP* segregants carrying the transgene were significantly more resistant to Δ*raxX(raxX*) with approximately 8 cm average lesion lengths compared to null segregants that developed significantly longer average lesion lengths of approximately 17 cm (Fig. S3). These results indicate that the XA21:EFR:GFP protein perceives RaxX.

For subsequent experiments, we focused on two *Xa21:EFR:GFP* lines (-2 and -6) for further molecular characterization. For these experiments, *T*_1_ plants were used for line 2 and *T*_2_ plants were used for line 6 (we self-pollinated *T*_1_ individuals and collected *T*_2_ seed for line 6) to test if similar phenotypes are observable in different lines and in subsequent generations. We found that *T*_2_ individuals from line 6 maintained *Xoo* resistance that segregated with the *Xa21:EFR:GFP* transgene (Fig. 1). Because *T*_1_ and *T*_2_ individuals from lines 2 and 6, respectively, were still segregating for the transgene, we performed experiments on individual plants that carried the *Xa21:EFR:GFP* transgene, selected by PCR genotyping. We used null segregant individuals as controls.

### The *Xa21:EFR:GFP* chimeric transgene is expressed and XA21:EFR:GFP protein accumulates in stable transgenic lines

**Figure 2:**
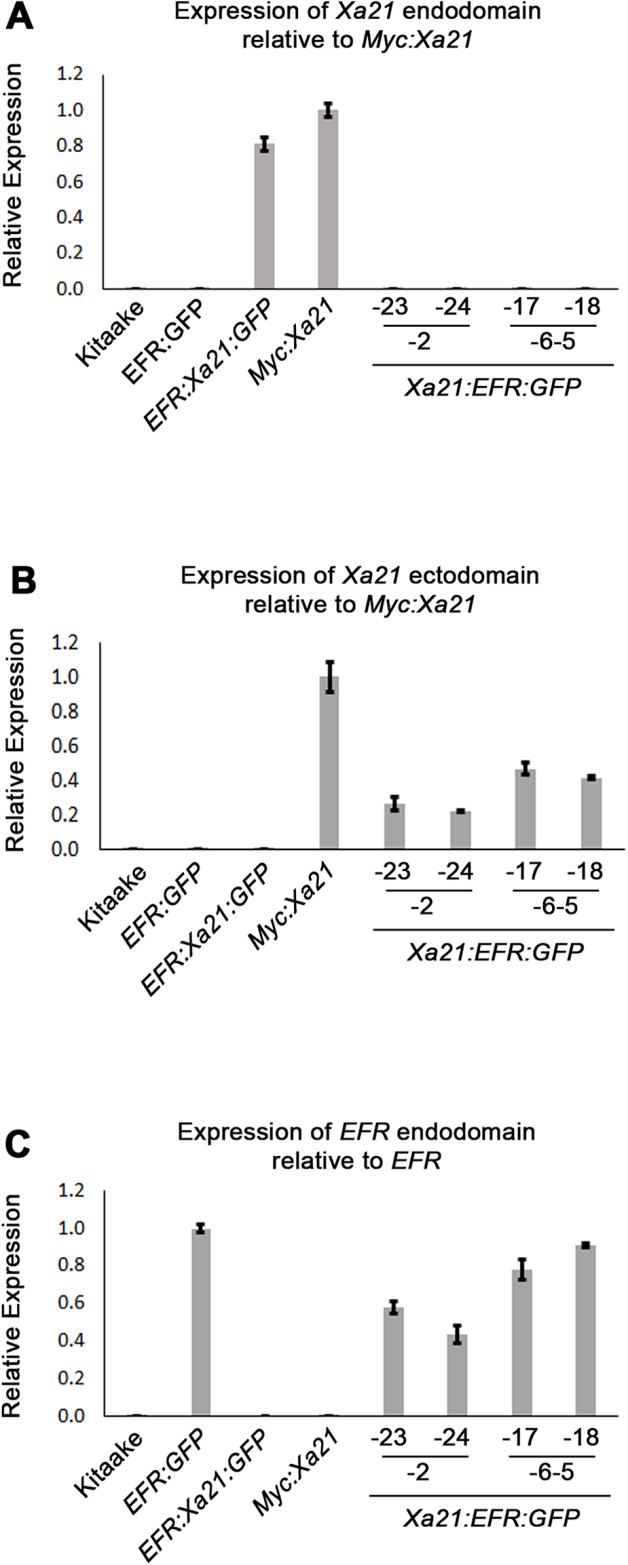
*Xa21:EFR:GFP* transcripts are expressed in stable transgenic lines. Bar graphs represent the relative expression of transgenic transcripts. (A) Relative amplification of the *Xa21* endodomain with *Myc:Xa21* rice as the expression reference. (B) Amplification of the *Xa21* endodomain with *Myc:Xa21* rice as the expression reference. (C) Amplification of the *EFR* cytoplasmic domain with *EFR:GFP* rice as the expression reference. Gene expression was measured by quantitative real-time PCR using cDNA amplified from total RNA as a template. Each gene expression measurement is the average of two technical replicates and error bars represent the standard deviation between the two measurements.

We used qRT-PCR to assess if plants containing the *Xa21:EFR:GFP* transgene express the *Xa21* ectodomain and *EFR* cytoplasmic domain. We assessed transcript levels using domain-specific primers for regions that encode the XA21 ectodomain, XA21 cytoplasmic domain, and the EFR cytoplasmic domain (Table S1) (Figs. 2A–2C). Our results show *Xa21:EFR:GFP*-2-23, -2-24, -6-5-17, and -6-5-18 that carry the transgene specifically express regions encoding the XA21 ectodomain and the EFR cytoplasmic domain. Additionally, these plants do not express regions encoding the XA21 endodomain. Because these plants are not expressing the full-length *Xa21* transcript or endodomain, any immune responses observed in these plants are not mediated by full-length XA21 or the XA21 endodomain.

In addition to the specific *Xa21:EFR:GFP* transcript, we show that XA21:EFR:GFP protein accumulates in transgenic rice. We performed Western Blot analysis to determine if XA21:EFR:GFP protein accumulates in *Xa21:EFR:GFP* transgenic rice using primary anti-GFP antibodies. Our results show that XA21:EFR:GFP protein is detectable in *Xa21:EFR:GFP*-2-28, -2-29, -6-5-4, and -6-5-7 that carry the *Xa21:EFR:GFP* transgene. Wild-type Kitaake and null segregants *Xa21:EFR:GFP*-2-32 and *Xa21:EFR:GFP*-6-5-6 do not express any GFP tagged protein (Fig. S3). Together, RNA and protein expression indicate that two independent *Xa21:EFR:GFP* transgenic lines express *Xa21:EFR:GFP* transcript and accumulate XA21:EFR:GFP protein.

### RaxX21-sY treated *Xa21:EFR:GFP* rice leaves produce reactive oxygen species and highly express stress-related genes

**Figure 3:**
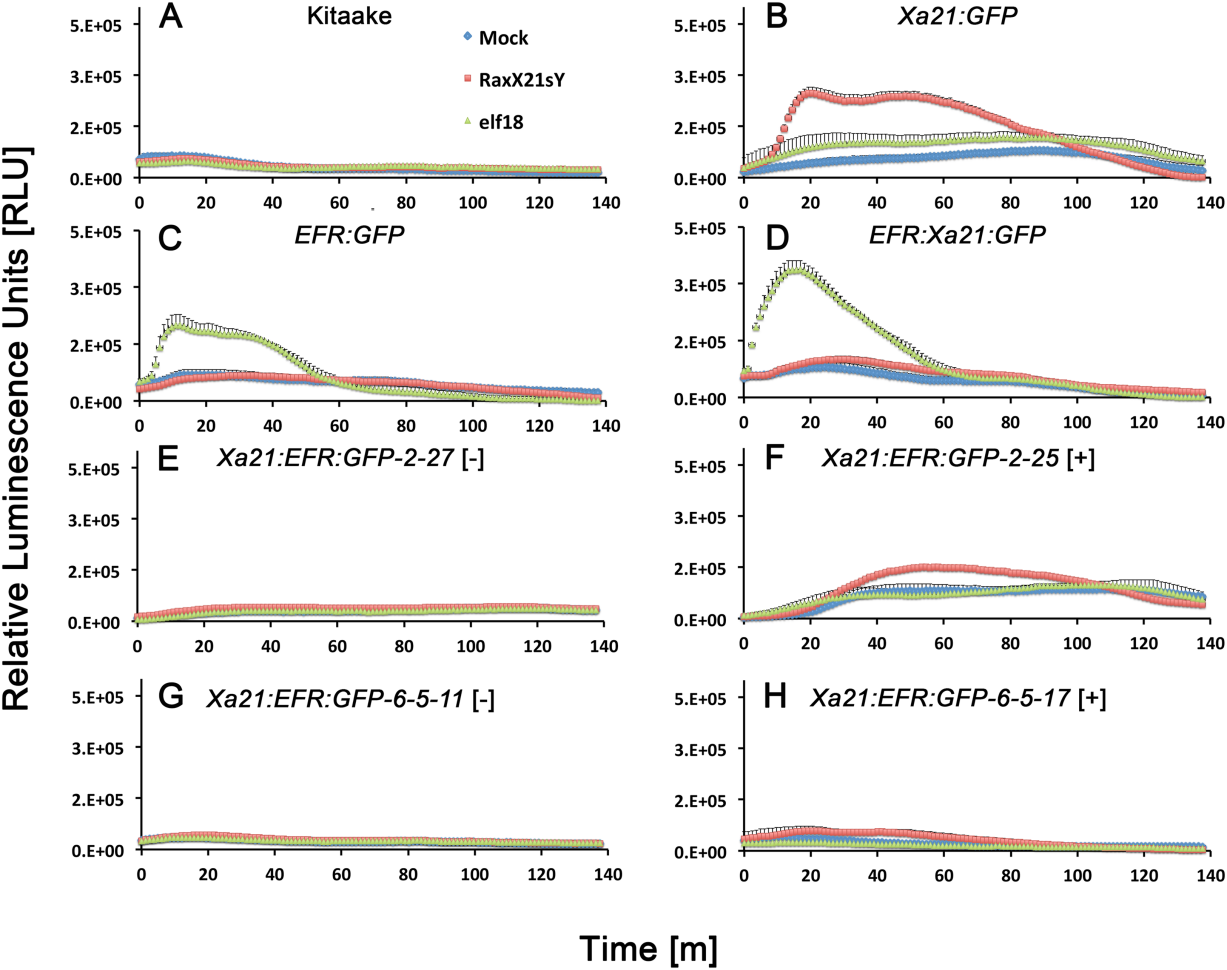
Reactive oxygen species accumulate after peptide treatments. Reactive oxygen species (ROS) production after water (mock, blue diamonds), 500 nM RaxX21-sY (red squares), or 500 nM elf18 peptide treatments (green triangles). (A) ROS production in wild-type Kitaake rice and (B) Xa21:GFP rice, (C) EFR:GFP rice and (D) EFR:Xa21:GFP rice. (E) and (G) ROS production in T1 and T2 null-segregant individuals from Xa21:EFR:GFP line -2 and line -6-5, respectively. (F) and (H) ROS production in T1 and T2 ndividuals from line -2 and -6-5, respectively, that segregate for the *Xa21:EFR:GFP* transgene. Each datapoint represents an average of four technical replicate measurements and error bars represent the standard error of the averages. These experiments have been repeated three times with similar results.

We next assessed if *Xa21:EFR:GFP* rice are able to activate immune responses after RaxX treatments. We used a commercially synthesized, sulfated RaxX peptide, composed of 21 amino acids from the *Xoo* RaxX protein sequence in PXO99A (RaxX21-sY) previously shown to activate XA21-mediated immunity (Pruitt et al., 2015; Wei et al., 2016). Bursts of ROS are commonly measured to assess immune responses because ROS are rapidly produced as a defense response to pathogen attack (Wojtaszek, 1997; Jones & Dangl, 2006; Macho & Zipfel, 2014). We therefore measured ROS production in *Xa21:EFR:GFP* rice after RaxX21-sY treatment to determine if plants carrying the chimeric protein respond similarly to RaxX21-sY treated plants carrying full-length XA21 (Pruitt et al., 2015). *Xa21:EFR:GFP* rice accumulate ROS in response to RaxX21-sY treatments, but not to mock or elf18 treatments (Fig. 3). In addition, we confirmed that RaxX21-sY treated XA21:GFP rice, expressing the full-length XA21 protein tagged with GFP (Fig. S4), accumulate ROS (Fig. 3B). Null segregants did not produce ROS bursts in response to RaxX21-sY treatments (Fig. 3). *EFR:GFP* and *EFR:XA21:GFP* rice responded to elf18, but not to RaxX21-sY, showing that the XA21 ectodomain in full-length XA21 and XA21:EFR:GFP proteins is necessary for RaxX-triggered immune responses (Figs. 3C and 3D).

**Figure 4:**
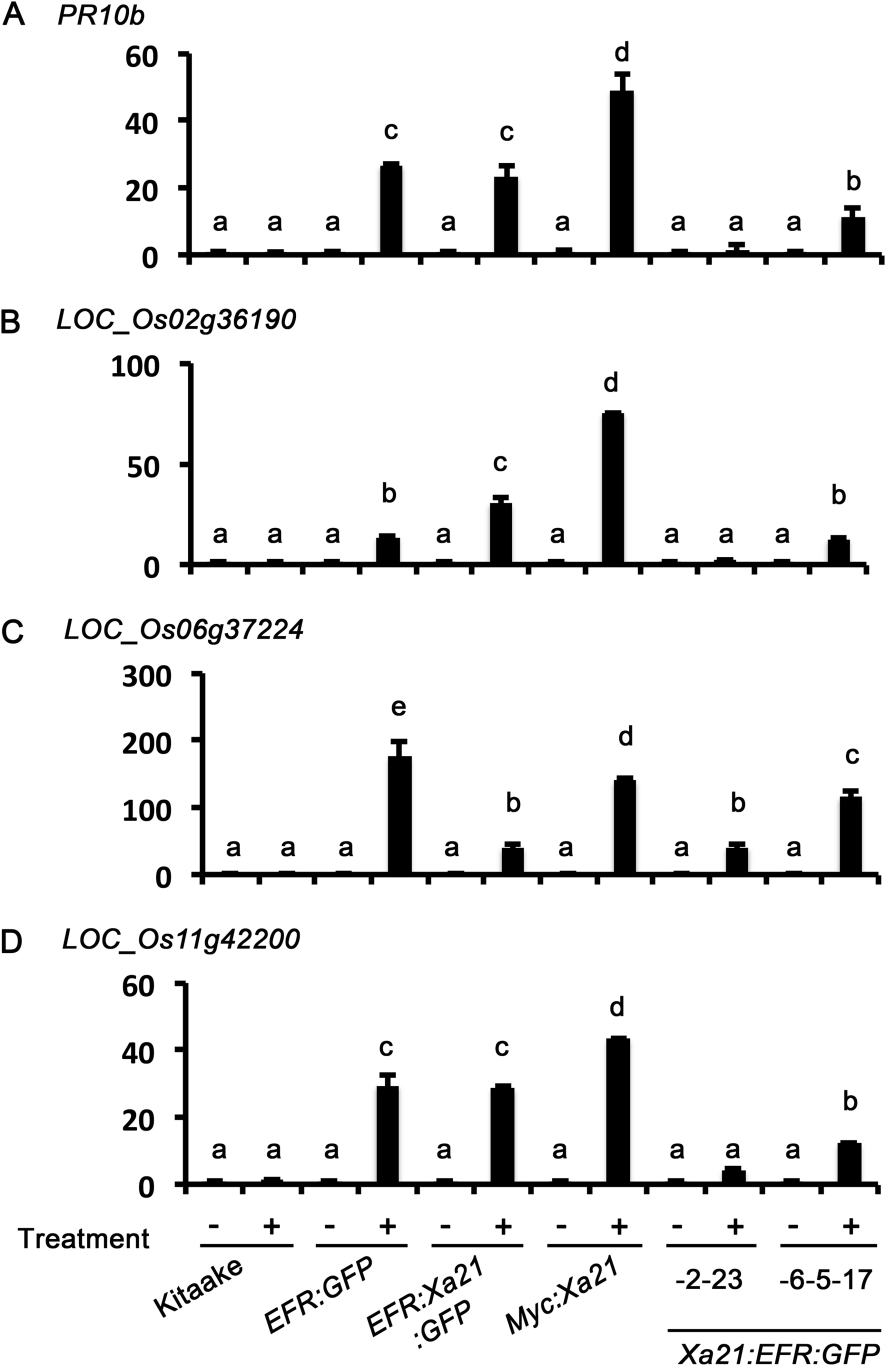
*Xa21:EFR:GFP* rice express stress-related genes after RaxX21-sY treatment. Gene expression profiles of four stress-related genes, (A) *PR10b*, (B) *LOC_Os2g36190*, (C) *LOC_06g37224*, and (D) *LOC_Os11g42200*. Samples are rice leaves from wild-type Kitaake, *Myc:XA21* rice, and individuals -23 from *Xa21:EFR:GFP* line -2 and individual -17 from line -6-5. Leaves were mock treated with water (-) or with 500 nM RaxX21-sY for *Myc:XA21* and *XA21:EFR* rice and 500 nM elf18 for *EFR:GFP* and *EFR:XA21:GFP* rice (+). Kitaake was treated with both RaxX21-sY and elf18 in these experiments. Letters indicate significant difference in gene expression compared to mock using the Tukey–Kramer HSD test (α = 0.05). Expression levels are normalized to mock treatment of the same line. Bars depict average expression level relative to actin expression ± standard error of three technical replicates. This experiment was repeated twice with similar results.

We next measured stress-related marker gene expression in RaxX21-sY treated *Xa21:EFR:GFP* rice to further characterize the XA21:EFR:GFP-mediated response. We measured the expression of rice defense marker genes *PR10b*, *LOC_Os02g36190*, *LOC_Os06g37224*, and *LOC_Os11g42200. PR10b* encodes a putative ribonuclease and is up-regulated upon fungal infection in rice (McGee, Hamer & Hodges, 2001), *LOC_Os02g36190* is involved in phytoalexin biosynthesis and bacterial blight resistance (Li et al., 2013), *LOC_Os06g37224* encodes an ent-Kaurene Oxidase up-regulated after ultraviolet light stress (Itoh et al., 2004), and *LOC_Os11g42200* encodes a laccase precursor protein and is up-regulated after Atrazine herbicide treatment (Huang et al., 2016). The up-regulation of these genes were previously established as markers of the rice stress-response including the XA21-mediated immune response to sulfated RaxX peptides and to *Xoo* infection (Chen et al., 2014; Pruitt et al., 2015; Thomas et al., 2016) using a detached leaf treatment assay. RNA was extracted from detached leaves of four-week-old plants mock treated with water or with 500 nM of RaxX21-sY for 6 h. Gene expression was measured in individuals *Xa21:EFR:GFP-2-23* and *-6-5-17* by quantitative real-time PCR. Higher expression was observed in each of the stress-related genetic markers in RaxX21-sY treated *Myc:Xa21* and *Xa21:EFR:GFP* rice (Figs. 4A–4D). Gene expression was not induced in any mock treated samples or Kitaake samples treated with RaxX21-sY. Only Individual 2–23 showed higher induction of *LOC_Os11g42200* and *LOC_Os06g37224*, and only induction of *LOC_Os06g37224* was significant (Fig. 4C). Although the magnitude of ROS and stress gene induction in response to RaxX21-sY is relatively lower in *Xa21:EFR:GFP* rice compared to *XA21:GFP* rice (Figs. 3 and 4), both show robust resistance to *Xoo* infection as measured by lesion length progression (Fig. 1) Together, the results from ROS and gene expression experiments suggest that the XA21 ectodomain in XA21:EFR:GFP is sufficient to recognize RaxX and that the EFR endodomain can be substituted for the XA21 endodomain to transduce immune responses after RaxX treatment.

## Discussion

Here we show that the ectodomain of XA21 is sufficient to confer full resistance to *Xoo* strain PXO99A when fused to the intracellular domain of the *Arabidopsis* immune receptor EFR (Fig. 1 and Figs. S1–S3). We previously demonstrated that a functional EFR:XA21:GFP is not able to confer resistance to *Xoo* when expressed in rice. Together these results suggest that the XA21 extracellular domain and the recognition of RaxX are the key properties that dictate the robust immune response of XA21. Both the native XA21 endodomain as well as the EFR endodomain fused to the XA21 ectodomain appear to be interchangeable as both XA21 and EFR kinases can confer robust resistance when fused with the XA21 ectodomain. This result slightly contrasts with previous domain swap studies that indicated that the endodomains of immune receptors were the defining properties of the immune receptor responses (He et al., 2000; Brutus et al., 2010; Albert et al., 2010; Kishimoto et al., 2010). Although XA21 mutants and XA21 derivatives that lack a functional kinase domain maintain partial resistance, it appears that a functional kinase domain is required for robust resistance (Wang et al., 1998; Andaya & Ronald, 2003).

Despite the evidence that rice expressing XA21:EFR:GFP are resistant to *Xoo*, it is unclear why plants expressing the reciprocal EFR:XA21:GFP protein are susceptible to *Xoo* (Schwessinger et al., 2015a). Previous results indicate that elf18 and RaxX21-sY have similar EC_50_ values for immune activation (Schwessinger et al., 2015a; Pruitt et al., 2015), but it is unclear if RaxX and EF-Tu are available at similar levels during infection, and it is also possible that *Xoo* masks EF-Tu, preventing optimal EFR ectodomain recognition. We hypothesize that the XA21 ectodomain is critical for conferring robust resistance because it interacts with additional rice-specific signaling components that the EFR ectodomain is unable to bind. In partial support of this hypothesis, we previously showed that the EFR kinase domain does not interact with some of the previously identified XA21 kinase domain signaling components, including the negative regulator XB15 and positive regulator XB3 (Schwessinger et al., 2015a). It is therefore possible that unidentified positive regulators of XA21-mediated immunity that interact with the XA21 ectodomain do not associate with the EFR ectodomain. Future studies might be aimed at identifying these elusive ectodomain-specific signaling partners to better understand XA21-mediated immunity.

## Supplemental Information

10.7717/peerj.4456/supp-1 Supplemental Information 1 Inoculation of *Xa21:EFR:GFP* T_0_ lines with *Xoo* strain PXO99A. Average lesion lengths from ten independent T_0_ *Xa21:EFR:GFP* lines inoculated with *Xoo* PXO99A. Kitaake and XA21-Kitaake (XA21) rice were used as susceptible and resistant controls (green bars) and inoculated at the 5-week old stage. Transgenic *Xa21:EFR:GFP* rice were inoculated using the scissor clipping method approximately 4–5 weeks after regeneration. Plants were scored 14 days post inoculation. Error bars represent standard deviation of the mean lesion length measured from multiple leaves from the same plant (n ≥ 3). Statistical analysis was performed using the Tukey-Kramer HSD test for each individual experiment. Different letters indicate significant differences between averages (alpha = 0.05).

10.7717/peerj.4456/supp-2 Supplemental Information 2 Inoculation of *Xa21:EFR:GFP* T_1_ plants with *Xoo* strain PXO99A. T_0_ progeny from lines 2, 4, 5, 6 and 7 were inoculated with *Xoo* strain PXO99A. Black bars indicate T_1_ progeny that carry the *Xa21:EFR:GFP* transgene while red bars indicate null segregants. Kitaake, *EFR:GFP*, and *EFR:Xa21:GFP* rice controls are represented by green bars. +/− represents presence or absence of the *Xa21:EFR:GFP* transgene determined by PCR. Plants were inoculated at the 5 week-old stage and lesions were scored 14 days post inoculation. Control mean lesion lengths are calculated from pooled lesion measurements (n ≥ 9) from multiple plants and error bars for controls represent the standard deviation. Error bars for experimental samples represent standard deviation of the mean lesion lengths measured from multiple leaves (n ≥ 3) from the same plant. Statistical analysis was performed using the Tukey-Kramer HSD test for each individual experiment. Different letters indicate significant differences between means (alpha = 0.05).

10.7717/peerj.4456/supp-3 Supplemental Information 3 Inoculation of *Xa21:EFR:GFP* T_1_ and T_2_ progeny with *Xoo* Δ*raxX* strains and complemented Δ*raxX(raxX)* strains. T_1_ progeny from line -2-13, -2-19 and T_2_ progeny from line -6-5-1 were inoculated with *Xoo* strains PXO99A, a PXO99A with a deletion in the *raxX* gene (Δ*raxX*), and Δ*raxX* strains complemented with *raxX* (Δ*raxX(raxX)*). Plants were inoculated at the 5 week-old stage and lesions were scored 14 days post inoculation. Black bars indicate average lesion length measurements (n > 7) from multiple plants that carry the *Xa21:EFR:GFP* transgene. Red bars indicate average measurements (n ≥ 3) from single null segregant individuals infected with *ΔraxX(raxX)*. Green bars represent control average lesion length measurements (n ≥ 4) pooled from multiple Kitaake and *Xa21:GFP* rice plants. Error bars represent standard deviation. Asterisks (*) represent statistically significant differences between the indicated infections using the student’s T-test (*p* < 0.001). These experiments were repeated twice with similar results.

10.7717/peerj.4456/supp-4 Supplemental Information 4 *Xa21:EFR:GFP* rice containing the transgene express GFP-tagged chimeric protein. Western blot showing protein level of EFR:GFP, EFR:XA21:GFP, XA21:GFP, and XA21:EFR:GFP using an anti-GFP antibody to detect the C-terminal GFP tag. The lower panel shows the coomassie brilliant blue staining of the membrane as a loading control. + and − indicates the presence of the transgene determined by PCR. XA21:EFR:GFP samples were from T1 individuals *XA21:EFR:GFP*-2-28, -2-29, and -2-32 and T2 individuals *XA21:EFR:GFP*-6-5-4, -6-5-6, and -6-5-7.

10.7717/peerj.4456/supp-5 Supplemental Information 5 List of primers used in this study. Table indicates each primer used in this study. The first column shows the 5′ to 3′ primer sequence. The second column indicates the associated MSU locus ID, primer direction, and purpose of primer. The last column indicates figures associated with each primer set.

## Additional Information and Declarations

### Data Availability

The following information was supplied regarding data availability: The data are represented in the Figures and Supplemental Files.
