# Supplementary figures and images for: The rice XA21 ectodomain fused to the Arabidopsis EFR cytoplasmic domain confers resistance to Xanthomonas oryzae pv. oryzae
Source: PeerJ. 2018 May 9;6:e4456. doi: 10.7717/peerj.4456 (PMC5949059; doi:10.7717/peerj.4456)

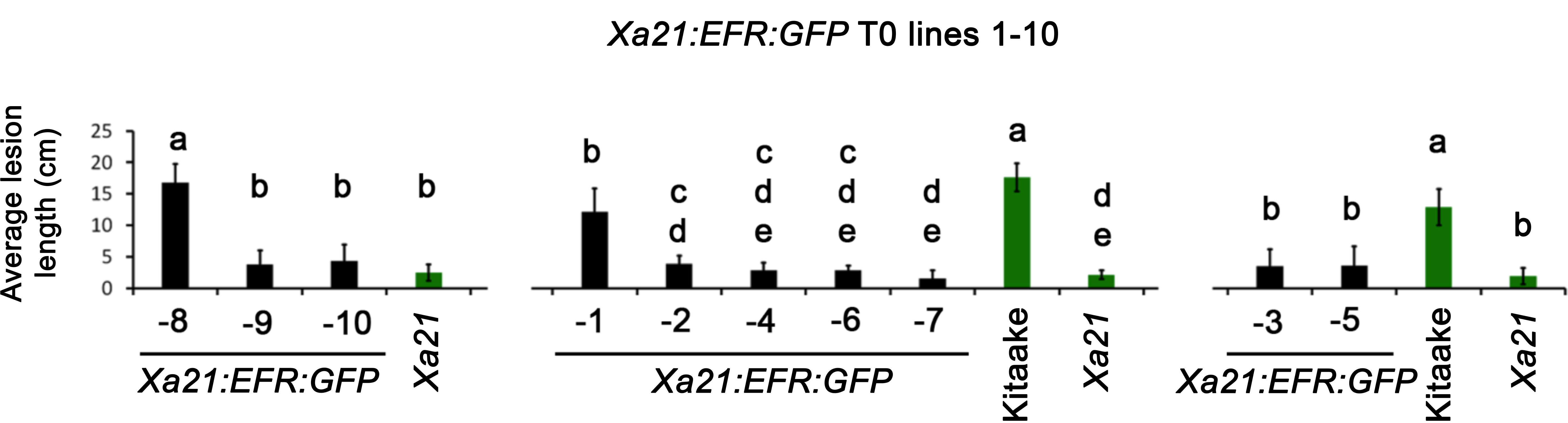

Supplement: Supplemental Information 1 — Average lesion lengths from ten independent T0 Xa21:EFR:GFP lines inoculated with Xoo PXO99A. Kitaake and XA21-Kitaake (XA21) rice were used as susceptible and resistant controls (green bars) and inoculated at the 5-week old stage. Transgenic Xa21:EFR:GFP rice were inoculated using the scissor clipping method approximately 4–5 weeks after regeneration. Plants were scored 14 days post inoculation. Error bars represent standard deviation of the mean lesion length measured from multiple leaves from the same plant (n ≥ 3). Statistical analysis was performed using the Tukey-Kramer HSD test for each individual experiment. Different letters indicate significant differences between averages (alpha = 0.05). [file peerj-06-4456-s001.png]

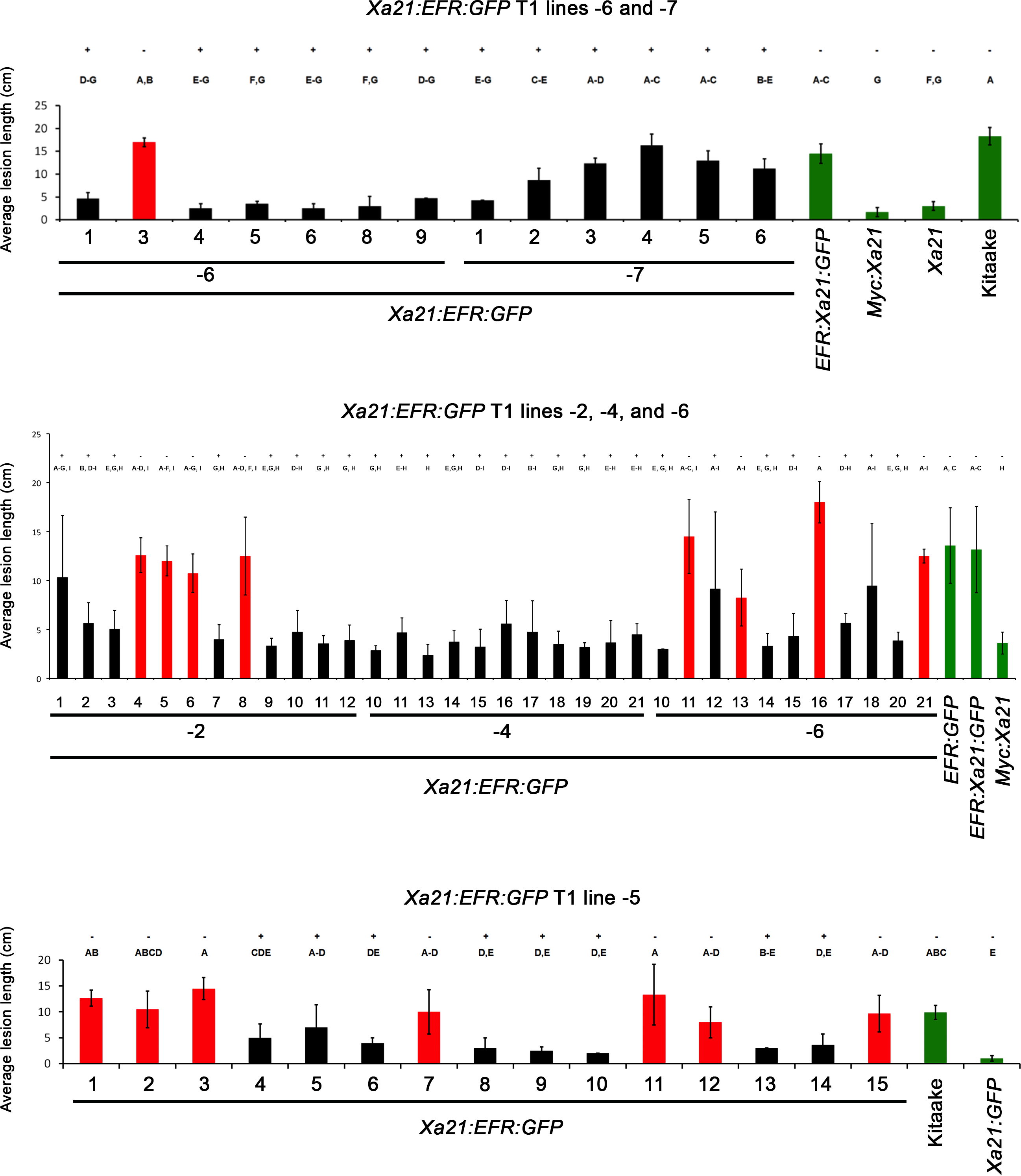

Supplement: Supplemental Information 2 — T0 progeny from lines 2, 4, 5, 6 and 7 were inoculated with Xoo strain PXO99A. Black bars indicate T1 progeny that carry the Xa21:EFR:GFP transgene while red bars indicate null segregants. Kitaake, EFR:GFP, and EFR:Xa21:GFP rice controls are represented by green bars. +/− represents presence or absence of the Xa21:EFR:GFP transgene determined by PCR. Plants were inoculated at the 5 week-old stage and lesions were scored 14 days post inoculation. Control mean lesion lengths are calculated from pooled lesion measurements (n ≥ 9) from multiple plants and error bars for controls represent the standard deviation. Error bars for experimental samples represent standard deviation of the mean lesion lengths measured from multiple leaves (n ≥ 3) from the same plant. Statistical analysis was performed using the Tukey-Kramer HSD test for each individual experiment. Different letters indicate significant differences between means (alpha = 0.05). [file peerj-06-4456-s002.png]

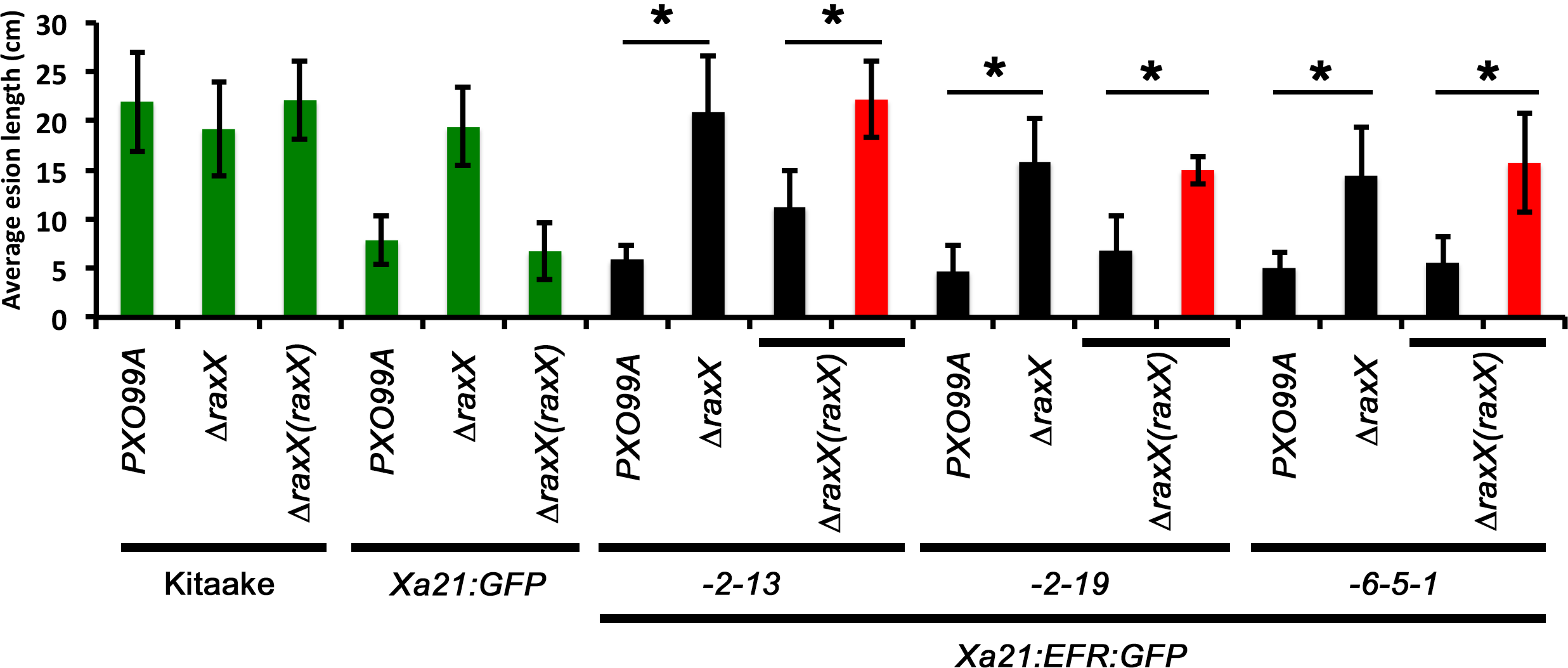

Supplement: Supplemental Information 3 — T1 progeny from line -2-13, -2-19 and T2 progeny from line -6-5-1 were inoculated with Xoo strains PXO99A, a PXO99A with a deletion in the raxX gene (ΔraxX), and ΔraxX strains complemented with raxX (ΔraxX(raxX)). Plants were inoculated at the 5 week-old stage and lesions were scored 14 days post inoculation. Black bars indicate average lesion length measurements (n > 7) from multiple plants that carry the Xa21:EFR:GFP transgene. Red bars indicate average measurements (n ≥ 3) from single null segregant individuals infected with ΔraxX(raxX). Green bars represent control average lesion length measurements (n ≥ 4) pooled from multiple Kitaake and Xa21:GFP rice plants. Error bars represent standard deviation. Asterisks (*) represent statistically significant differences between the indicated infections using the student’s T-test (p < 0.001). These experiments were repeated twice with similar results. [file peerj-06-4456-s003.png]

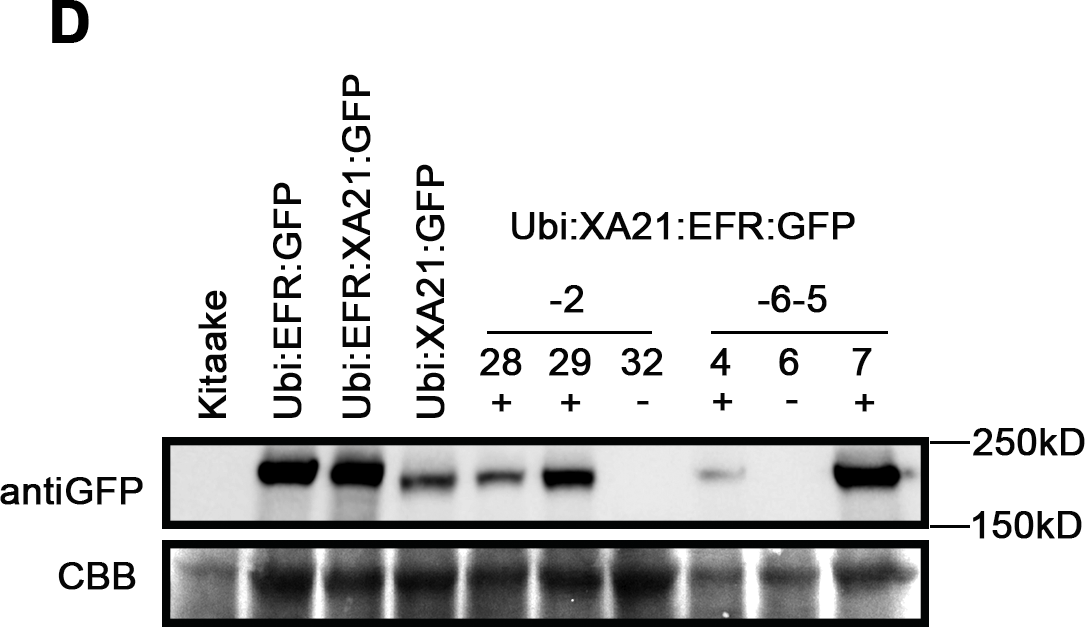

Supplement: Supplemental Information 4 — Western blot showing protein level of EFR:GFP, EFR:XA21:GFP, XA21:GFP, and XA21:EFR:GFP using an anti-GFP antibody to detect the C-terminal GFP tag. The lower panel shows the coomassie brilliant blue staining of the membrane as a loading control. + and − indicates the presence of the transgene determined by PCR. XA21:EFR:GFP samples were from T1 individuals XA21:EFR:GFP-2-28, -2-29, and -2-32 and T2 individuals XA21:EFR:GFP-6-5-4, -6-5-6, and -6-5-7. [file peerj-06-4456-s004.png]
